# Sensitive and Specific TaqMan Real-Time PCR Assay for Beak and Feather Disease Virus in Psittacine Birds

**DOI:** 10.3390/vetsci12121153

**Published:** 2025-12-03

**Authors:** Bruno Fernandes, Teresa Fagulha, Sílvia Carla Barros, Fernanda Ramos, Tiago Luís, Ana Duarte, Margarida Dias Duarte, Ana Margarida Henriques

**Affiliations:** 1 Laboratório de Virologia, Instituto Nacional de Investigação Agrária e Veterinária, 2780-157 Oeiras, Portugal; brufe@samf.ku.dk (B.F.); teresa.fagulha@iniav.pt (T.F.); silvia.santosbarros@iniav.pt (S.C.B.); fernanda.ramos@iniav.pt (F.R.); tiago.luis@iniav.pt (T.L.); ana.duarte@iniav.pt (A.D.); margarida.duarte@iniav.pt (M.D.D.); 2 Department of Public Health, Center for Health Services Research, University of Copenhagen, Øster Farimagsgade Build. 24 Q, 1st Floor, 1353 Copenhagen K, Denmark; 3 Faculdade de Ciências e Tecnologia, Departamento de Ciências da Vida, Universidade Nova de Lisboa, Campus da Caparica, 2829-516 Caparica, Portugal; 4 Centre for Interdisciplinary Research in Animal Health (CIISA), Faculty of Veterinary Medicine, University of Lisbon, 1300-477 Lisbon, Portugal; 5 Associate Laboratory for Animal and Veterinary Sciences (AL4AnimalS), Faculty of Veterinary Medicine, University of Lisbon, 1300-477 Lisbon, Portugal

**Keywords:** beak and feather disease virus, circovirus, psittacine birds, real-time PCR, TaqMan probe

## Abstract

Beak and feather disease virus (BFDV) is a small, icosahedral, non-enveloped virus with a circular single-stranded DNA genome, that belongs to the *Circoviridae* family. BFDV is globally distributed and poses a major threat to susceptible avian populations. This underscores the urgent need for rapid and reliable molecular detection methods to monitor and control the spread of the virus. Quantitative PCR assays offer several advantages, including high sensitivity, specificity, and the ability to quantify viral load. Here, we report the development and validation of a quantitative real-time PCR assay using a TaqMan probe targeting the replicase gene to detect BFDV in psittacine blood samples. The assay demonstrates high sensitivity, specificity, and suitability for routine diagnostic use with an LOD and an LOQ of 10 and 30 plasmid copies, respectively, determined using a recombinant plasmid containing a BFDV genomic fragment. The assay achieved 98.8% sensitivity and 100% specificity. The method also demonstrated strong repeatability and reproducibility, with intra- and inter-assay coefficients of variation of 4.14% and 4.44%, respectively. Both values are well below the acceptance threshold. Overall, this TaqMan-based real-time PCR assay is a reliable, sensitive, and efficient diagnostic tool for detecting BFDV in psittacine samples.

## Simple Summary

Beak and feather disease virus (BFDV) is a harmful virus that affects parrots and related birds, damaging their feathers and beaks and weakening their immune systems. It has spread worldwide and poses a serious risk to bird populations, making fast and accurate detection very important. In this study, a real-time PCR, which can quickly identify the presence of the virus in psittacine blood samples was developed and validated. The test was shown to be both highly sensitive and highly specific. In trials using almost 100 bird samples, the method successfully identified nearly all infected cases with 98.8% accuracy, while never misidentifying uninfected birds. The test also produced consistent results when repeated, proving its reliability for routine use. Overall, this new detection tool will help veterinarians and scientists monitor and control the spread of BFDV more effectively, supporting efforts to protect vulnerable bird species and improve conservation strategies.

## 1. Introduction

The Beak and Feather Disease Virus (BFDV) is the causative agent of a disease named Psittacine Beak and Feather Disease (PBFD), that affects psittacine birds and is characterized by feather dystrophy, beak deformities, and claw abnormalities. BFDV belongs to the genus *Circovirus* within the family *Circoviridae*, which includes some of the smallest known viruses [1,2]. Members of this genus are non-enveloped, icosahedral viruses that contain a single-stranded circular DNA genome of approximately 2000 nucleotides, with a diameter of about 15–26 nm [3,4].

BFDV was first described in Australian cockatoos in the 1970s [5]. Nowadays, PBFD is now one of the most significant viral diseases of psittacine birds worldwide [6,7,8], mainly due to both legal and illegal international trade of exotic parrots, that contributed to the global dissemination of the virus. Recent epidemiological studies have detected BFDV in wild and captive populations on nearly every continent [9,10], raising significant concerns for biodiversity conservation.

All psittacine species are considered susceptible to BFDV, with infections reported in over 60 species of wild and captive parrots and cockatoos [2,11]. The disease is officially recognized as a key threatening process for endangered psittacine birds in Australia (Department of Agriculture, Water and the Environment, 2020) and has also been reported as a conservation threat in other regions such as Africa [8] and South America [12].

The virus exhibits tropism for mitotically active cells in the beak and feather epithelia of psittacine birds, and to lymphoid tissues causing severe malformations and immunosuppression. Avian circovirus infections exhibit a wide clinical spectrum, ranging from non-specific signs such as lethargy, ill-thrift, and growth retardation to chronic, severe integumentary lesions involving the feathers, beak, and claws. A consistent pathological feature of PBDF is lymphoid tissue depletion, which leads to immunosuppression and increased susceptibility to secondary opportunistic infections [3,4,13]. Viral loads may persist in asymptomatic birds, indicating the presence of subclinical infections that serve as cryptic reservoirs for viral transmission [7,14].

A rapid and accurate diagnosis of BFDV is of the utmost importance in order to guide clinical decision-making and welfare management, as well as to prevent outbreaks by identifying subclinical carriers who are spreading the virus silently. The most reliable diagnostic method is the amplification of BFDV-DNA by PCR. Several methods are described in the literature, including the conventional PCR assays described by Ypelaar [15] and Mankertz [16]. Since then, some intercalating dye-based real-time PCR assays were described [17,18,19]. To our knowledge, only Cerníková and colleagues [20] are the only authors to have described a validated TaqMan real-time PCR assay for BFDV diagnosis. This assay targets a conserved replicase-encoding gene region and demonstrates a good analytical performance. However, additional methods are still needed, as reliance on a single genomic target makes the assay vulnerable to sequence variability, sample degradation, and emerging viral mutations which may reduce detection efficiency.

This paper reports the development and validation of a real-time PCR using a TaqMan probe to detect BFDV in psittacine samples, using primers and a probe directed towards a conserved region of the replicase-encoding gene.

## 2. Materials and Methods

### 2.1. Primers and Probe Design

A preliminary alignment was performed with approximately 30 sequences, representatives of the nine BFDV genotypes described by our group in 2010 [21]. The rep-encoding gene region was selected due to its lower diversity (92–94% nucleotide similarity) among the analyzed strains, compared to that of the cap-encoding gene (84–86%). (For the real-time PCR, primers BFDV-F and BFDV-R were selected, with sequences 5′-TTGTGGCGAGRGAYGGTC-3′ and 5′-TCTCGCGCGAYNTCYTTC-3′, respectively. These primers allow the amplification of an 85 bp conserved region of the rep gene. The probe used, with the sequence 5′-FAM-TCCDGHCATCACGGCRGCAAC-TAMRA-3′, was designated BFDV-P (Table 1). A recombinant plasmid TA-BFDV, was constructed as described below and used as a positive control for the reaction and as a reference strain for the validation assays.

The BFDV-P real-time PCR failed to detect one of the BFDV-positive samples used to validate the method. Positive amplification was confirmed by agar gel electrophoresis. Nucleotide sequence analysis of the rep-encoding gene from this specific BFDV sample revealed mutations in the probe annealing region, explaining the failure to detect the positive sample. Therefore, a second probe (BFDV-P2) with the sequence 5′-ROX-TCCDGHAAGCACAGCRGCAAC-BHQ2-3′ was designed (Table 1). Although this sequence appears to be rare, as no similar sequences are available in the databases, the method described here can be performed with the two probes simultaneously, maintaining the PCR conditions.

### 2.2. Real-Time PCR

Blood samples from psittacine birds, collected in EDTA-containing tubes, were previously received at INIAV for BFDV diagnosis by conventional PCR. The veterinarian responsible for these samples provided a formal declaration authorizing their use for scientific purposes, and therefore, samples testing BFDV-positive were stored for further studies. Eighty-three samples were randomly selected for extraction that was performed using a KingFisher Flex nucleic acid extraction workstation (ThermoFisher Scientific, Waltham, MA, USA), according to the manufacturer’s instructions. A dilution of 1:20 of EDTA-blood in a total volume of 200 µL PBS was performed prior to extraction. The extracted DNA was then used as a template in a real-time PCR. The reaction was performed with 500 ng of total DNA, 1 µM of each primer and 0.5 µM of each probe, using Speedy NZYTaq 2× Colourless Master Mix (Nzytech, Lisbon, Portugal), according to the manufacturer’s protocol. The amplification program included an initial denaturation step at 95 °C for 1 min, followed by 45 cycles of denaturation at 95 °C for 2 s and a combined annealing/extension step at 60 °C for 5 s.

The conventional PCR previously used to characterize the samples for validation in this study (as described by Mankertz et al. [16], targeting the *rep* protein gene), was performed using the NZYTaq II 2× Green Master Mix (Nzytech) with 500 ng of total DNA and 1 µM of primers (#176 and #177) in a final volume of 25 µL. The amplification program consisted of an initial denaturation step at 95 °C for 3 min, followed by 45 cycles of denaturation at 95 °C for 30 s, annealing at 49 °C for 30 s and extension at 72 °C for 30 s. A final extension step for 5 min at 72 °C was performed and the amplified product of 240 bp was observed in a 1% agarose gel, after a run of about 40 min at 120 V. NZYDNA Ladder V (100–1000 bp) (Nzytech) was used to verify product size.

### 2.3. Construction of a TA-BFDV Recombinant Plasmid

A recombinant TA-BFDV plasmid was constructed by cloning a BFDV amplified PCR product into the pCR2.1 vector, using the Original TA Cloning kit (Invitrogen, Waltham, MA, USA), according to the manufacturer’s instructions. Briefly, the PCR product obtained from a BFDV-positive field strain as described before was separated by 1% agarose gel electrophoresis. The band of interest was then excised and purified, using NZYGelpure kit (Nzytech). A ligation reaction was performed with 2 µL of the purified amplicon and 3 µL of the vector at a predicted molar ratio of (1 vector)–(1.5 insert) in a total volume of 10 μL for 30 min at room temperature. The transformation of the One Shot TOP10 Chemically Competent *E. coli* cells (Thermo Fisher Scientific) was performed according to the OneShot transformation protocol present in the TA Cloning Kit user manual (Invitrogen). Recombinant plasmids were selected by restriction analysis and real-time PCR. The correct construction of the TA-BFDV recombinant was confirmed by Sanger sequencing.

### 2.4. Determination of the Limit of Detection

The limit of detection is defined as the lowest number of copies of the target that can be detected in a real-time PCR. To determine this limit, the assay was performed using tenfold serial dilutions of the recombinant plasmid TA-BFDV. The concentration of TA-BFDV was determined by fluorometry using a Qubit 4 Fluorometer (Invitrogen, USA) to be 108 ng/μL, equivalent to 2.5 × 10^10^ plasmid copies/μL. A total of 4 μL were used in the real-time PCR, corresponding to 10^11^ plasmid copies. Twelve tenfold dilutions were prepared down to 10^−1^ copies of the plasmid, with each plasmid concentration tested in four replicates.

### 2.5. Repeatability and Reproducibility Evaluations

To evaluate the variation in results obtained by using this real-time PCR detection system, intra- and inter-assay variations were assessed by testing the recombinant plasmid TA-BFDV in replicates and in independent assays, respectively. Intra-assay variability was evaluated by testing nine replicates within the same experiment, using an identical reaction mixture for each replicate. Inter-assay variability, in turn, was assessed based on the Cq values obtained from six independent assays. In both cases, the coefficient of variation (%CV) was calculated as the ratio of the standard deviation to the mean Cq value.

### 2.6. Sensitivity and Specificity

The sensitivity of the method described here was evaluated by testing 83 DNA samples that were confirmed positive using the conventional PCR method described by Mankertz and colleagues [16].

To evaluate the specificity of the test, 13 samples that were confirmed negative by another PCR method [16] were tested. Specificity was also assessed by testing 10 samples that had previously been confirmed as positive for other avian viruses in our laboratory, namely avian influenza virus (AIV), Newcastle disease virus (NDV), infectious bronchitis virus (IBV), avian reovirus (ARV), infectious bursal disease virus (IBDV), avian poxvirus (APV), avian polyomavirus (APyV), avian bornavirus (ABV), Pacheco’s disease virus (PsHV1), and psittacid herpesvirus type 5 (PsHV5).

A statistical analysis was performed using an unpaired *t*-test with GraphPad Prism 5 Demo software. For the statistical analysis, the Cq value of the negative samples was considered to be Cq = 40, and a *p*-value below 0.05 was considered statistically significant.

## 3. Results

### 3.1. Primers and Probe Design

Representative sequences of all the available genotypes in GenBank were aligned and the conserved regions were selected to design the primers and a probe for the real-time PCR BFDV detection assay. Degenerated bases were designed for nucleotide positions comprising more than one nucleotide among the aligned sequences to allow the detection of any strain. However, of the 83 positive samples used, one BFDV-positive sample was not detected using this probe-based method. The analysis of the sequence of this sample corresponding to the probe annealing showed three nucleotide differences that can justify the failure observed. Despite this variant probably not representing the circulating isolates, a second probe (BFDV-P2) was designed to detect it. This probe can be used together with the primers and probe of the developed and validated method, since the probes are labeled with different fluorophores and quenchers (Table 1). The PCR conditions in the duplex format are the same described above. Cq values obtained as well as reaction efficiency are similar when only one or the two probes are included in the reaction.

The TA-BFDV recombinant plasmid used in the validation assays was derived from a field strain whose rep-encoding gene is complementary only to BFDV-P (labeled with the FAM reporter fluorophore). Thus, fluorescence was generated exclusively with probe BFDV-P despite reactions being conducted with both probes. Consequently, only the FAM signal is reported.

### 3.2. Determination of the Limit of Detection (LOD) and Limit of Quantitation (LOQ)

**Figure 1:**
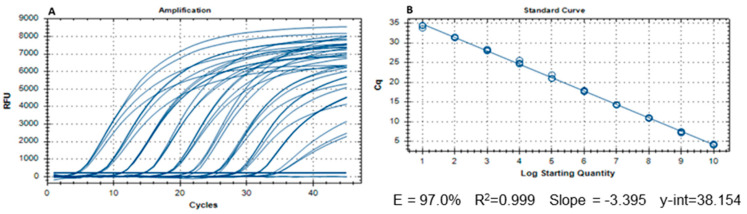
(**A**)—Cycle graph obtained in the BFDV real-time PCR performed for the limit of detection determination (from 1010 to 101 copy numbers). (**B**)—Standard calibration curve obtained by representing the obtained Cq values from different dilutions in function of the logarithm of the number of plasmid copies. “E” refers to reaction efficiency, while “R^2^” refers to coefficient of determination and y-int to the *y*-axis interception.

The recombinant plasmid TA-BFDV was tested using the real-time PCR method in 13 tenfold increments, ranging from 10^11^ to 10^1^ copies of the plasmid. The assay was performed in quadruplicate, and the obtained Cq values were recorded. The curves obtained in the real-time PCR are shown in Figure 1A. A calibration curve was obtained by plotting the Cq values against the log of the number of copies (Figure 1B). A correlation coefficient of 0.999 was obtained. Since the number of plasmid copies decreases as Cq increases, the slope is negative. For tenfold serial dilutions, the slope is −3.33 when E = 100%, and a perfect doubling of the number of DNA molecules occurs [22]. In this case, an acceptable slope of −3.395 was obtained, corresponding to a PCR efficiency of 97% (given by the expression $E=\left(10^{-(1/slope)}-1\right)\;*\;100$). DNA amplification was achieved in all dilutions except the two highest, indicating that the LOD corresponds to 10 plasmid copies, since it was the smallest concentration at which DNA was still detected. The LOQ is typically estimated as approximately three times the LOD, in this case, 30 plasmid copies.

### 3.3. Sensitivity and Specificity

Specificity is the proportion of negative samples that are correctly identified by the test. To evaluate the specificity of the PCR, 13 negative samples were assayed, all negative. Test’s specificity was also evaluated based on its ability to generate negative results with samples positive for other avian viruses. A total of 10 positive samples for other avian diseases viruses, namely avian influenza virus (AIV), Newcastle disease virus (NDV), infectious bronchitis virus (IBV), avian reovirus (ARV), infectious bursal disease virus (IBDV), avian poxvirus (APV), avian polyomavirus (APyV), avian bornavirus (ABV), Pacheco’s disease virus (PsHV1), and psittacid herpesvirus type 5 (PsHV5) tested negative in the PCR, which further demonstrates the test’s 100% specificity.

**Figure 2:**
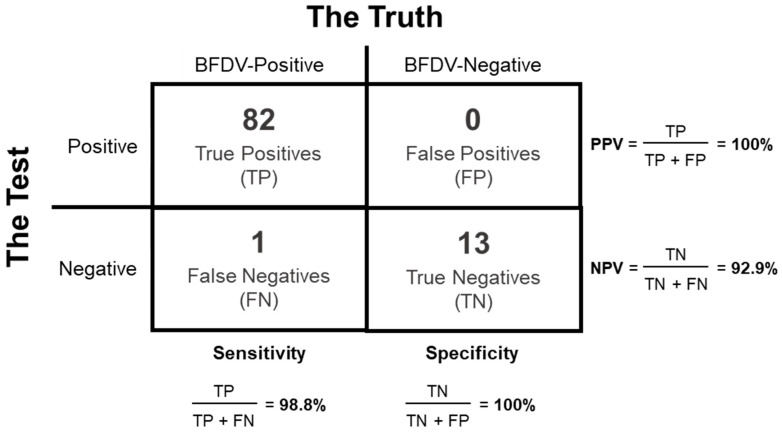
Matrix used for the determination of the sensitivity (98.8%), specificity (100%), positive predictive value (PPV) (100%) and negative predictive value (NPV) (92.9%) of the real-time PCR BFDV test, obtained by testing 83 positive and 13 negative BFDV samples.

The sensitivity of the test reported here was evaluated by measuring the proportion of positive samples that were correctly identified by the PCR. For this purpose, 83 DNA samples that tested positive for BFDV by a conventional PCR were assayed. Since 82 of the samples tested positive in this real-time PCR, the results indicate an overall sensitivity of 98.8% (Figure 2).

All samples, both negative and positive, were coded prior to analysis to guarantee that the results were generated under blinded conditions, minimizing potential bias.

Additionally, Positive Predictive Value (PPV) and Negative Predictive Value (NPV), were determined. The PPV is the proportion of positive test results that are true positives, and the NPV is the proportion of negative test results that are true negatives. The PPV was 100%, while the NPV was 92.9%, due to the failure to detect one positive sample (Figure 2).

**Figure 3:**
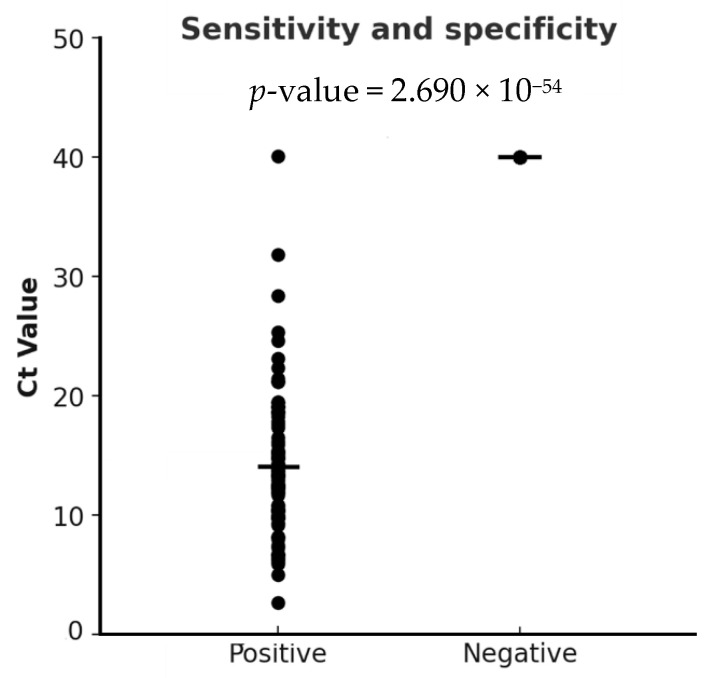
Comparison of Cq values obtained between positive and negative samples. Each dot represents an individual sample, and the horizontal bars indicate the mean value. For negative samples, a Cq = 40 was considered. A highly significant difference was observed between the groups (*p*-value = 2.690 × 10^−54^), demonstrating the high sensitivity and specificity of the test.

A Student’s *t*-test was performed to compare the Cq values obtained in the PCR test for positive (*n* = 83) and negative (*n* = 13) DNA samples (Figure 3). For negative samples, a Ct value of 40 (Cq = 40) was assumed. The analysis yielded a t-statistic of −38.53 and a corresponding *p*-value of 2.69 × 10^−54^ (*p* < 0.0001), indicating a significant difference between the two groups. The negative sign of the t-statistic reflects the lower mean Cq values observed in the positive group compared to the negative group. Given the magnitude of the test statistic and the exceedingly small *p*-value, the likelihood that this difference arises from random variation is negligible. These findings demonstrate robust separation of the Cq distribution between positive and negative samples, supporting the capacity of the assay to effectively discriminate between the two conditions.

### 3.4. Repeatability and Reproducibility Evaluations

The repeatability of the system was evaluated through intra-plate variability by testing nine replicates of the same sample containing the TA-BFDV recombinant plasmid in the same real-time PCR assay. The reproducibility of the system was assessed through an inter-plate variability evaluation, by testing the TA-BFDV recombinant plasmid in six independent real-time PCRs. In both cases, the evaluation was performed as a percentage of the coefficient of variation in the Cq values obtained. Table 2 shows the coefficients of variation obtained with the samples used in the intra- and inter-plate variability evaluations.

The coefficient of variation (CV) obtained in the intra-plate assay was 4.14%, while the inter-plate assay showed a CV of 4.44%.

## 4. Discussion

This paper reports the development and validation of a quantitative real-time PCR assay targeting the replicase gene to detect BFDV in psittacine DNA samples. Although a similar assay has already been described [20], additional methods are needed to address sequence variability and emerging viral mutations, thereby improving reliability and providing confirmatory testing options. A primers and probe set was designed for the development of a real-time BFDV detection assay, based on the alignment of representative sequences of all the available genotypes in GenBank. Both primers and probe sequences include degenerated bases for nucleotide positions comprising more than one nucleotide among the aligned sequences to allow the detection of any strain. Despite the use of degenerated bases, one BFDV-positive sample was not detected using this probe-based method. Analysis of the rep gene nucleotide sequence from this sample revealed differences in the nucleotide sequence compared to the probe sequence, explaining why this positive case was not detected. These specific nucleotide substitutions were not found in any other available GenBank sequence (23 September 2025), suggesting that they are rare. Of the three substitutions observed in this strain, two were non-synonymous, resulting in a substitution of methionine (ATG) for leucine (CTT). This amino acid substitution is unlikely to affect the replicase protein, as both residues possess hydrophobic side chains with similar nonpolar biochemical properties. The third substitution occurred in the third nucleotide position of the codon and therefore did not result in the substitution of the alanine residue (GCC → GCT). A second probe, labeled with different fluorophores and quenchers, was designed, and a duplex PCR able to detect all BFDV strains known until the moment was developed. Although this additional probe is necessary to guarantee the detection of all circulating strains, no other similar sequences were detected in the lab or in GenBank.

The sensitivity of the test can be evaluated by the ability of the method to detect positive samples, but also by the smallest number of viral copies that can be detected in the assay. This ability can be measured by both the limit of detection (LOD), that represents the lowest concentration at which the analyte can be reliably detected, and the limit of quantitation (LOQ), that corresponds to the lowest concentration that can be measured with acceptable precision and accuracy. The LOQ is typically estimated as approximately three times the LOD, reflecting a signal-to-noise ratio of about 10 compared to three for the LOD (European Commission, 2000) [23]. Reporting both parameters ensures accurate interpretation of results, since plasmid copies above 30 (LOQ) can be quantified reliably, while those between the 10 (LOD) and 30 (LOQ) should be considered detectable but not precisely measurable. Regarding analytical sensitivity, defined as the proportion of positive samples correctly identified by the PCR, the assay showed an overall sensitivity of 98.8% due to one failure among 83 positive samples. However, this failure was explained and subsequently resolved, allowing us to conclude that the test can be considered effectively 100% sensitive.

Also, the specificity of the test can be evaluated using two different indicators. Analytical specificity corresponds to the proportion of negative samples correctly identified by the test. The ability to generate negative results with samples positive for other avian viruses is also valuable. All 10 negative samples tested produced a negative result, as did positive samples for the other 10 avian viruses tested by this assay, indicating 100% specificity of the test. Of the positive samples for other viruses, five had a DNA genome and five had an RNA genome. In both cases, only the Taq DNA polymerase enzyme was used, and a PCR was performed. As the aim was to mimic real-life conditions, it would not have made sense to perform an RT-PCR.

The repeatability and reproducibility of the system was evaluated through intra-plate and inter-plate variabilities, respectively. In both cases, the evaluation was performed as a percentage of the coefficient of variation in the Cq values obtained. While the values obtained are similar, the inter-plate variability was higher than the intra-plate variability, as expected, since more factors can vary between each assay. As both values were lower than 5%, it can be concluded that the PCR is highly repeatable and reproducible.

Comparing the analytical performance of this method with that described by Cerníková and colleagues [20], it can be concluded that both assays exhibit comparable analytical sensitivity and specificity, with LODs of around 10 copies and excellent reproducibility. The present assay provides explicit LOQ determination and intra- and inter-assay variation data, offering additional information on quantitative reliability. Overall, both assays can be considered robust and suitable for routine diagnostic and surveillance use, with slight methodological distinctions reflecting their respective design and validation approaches.

## 5. Conclusions

In this study, we successfully developed and validated a quantitative real-time polymerase chain reaction (PCR) assay using a TaqMan probe to detect Beak and Feather Disease Virus in psittacine DNA samples. The assay demonstrated high analytical sensitivity, with a detection limit of 10 plasmid copies, and achieved 98.8% sensitivity and 100% specificity when evaluated against a diverse panel of positive and negative samples, including specimens infected with other avian viruses. Statistical analysis confirmed a highly significant distinction between the positive and negative groups, which further supports the diagnostic reliability of the assay. Additionally, the low coefficients of variation obtained in intra- and inter-assay tests highlight the robustness, repeatability, and reproducibility of the method. Taken together, these findings suggest that this assay is a reliable and efficient tool for BFDV detection, with potential applications in both clinical diagnostics and epidemiological surveillance. It may contribute to improved monitoring, control, and prevention strategies, thereby supporting the conservation of psittacine bird populations threatened by this virus.

## Figures and Tables

**Table 1:** Primer pair and TaqMan probe sequences designed for the detection of BFDV.

Primer/Probe	Sequence (5′ → 3′)	Position ^a^
BFDV-F	TTGTGGCGAGRGAYGGTC	436–453
BFDV-R	TCTCGCGCGAYNTCYTTC	520–503
BFDV-P	FAM-TCCDGHCATCACGGCRGCAAC-TAMRA	494–474
BFDV-P2	ROX-TCCDGHAAGCACAGCRGCAAC-BHQ2	494–474

^a^ Numbering according to BFDV sequence EU810208.

**Table 2:** Results of the intra-plate and inter-plate variability assays, in which nine replicates were tested in the same assay or six replicates were tested in separate independent assays, respectively. The Cq values obtained in each assay, as well the mean and standard deviation used for coefficient variation (CV (%)) determination are indicated.

	Intra-Plate Variability	Inter-Plate Variability
Replicate 1	12.65	11.32
Replicate 2	12.27	10.67
Replicate 3	12.24	11.52
Replicate 4	11.74	12.07
Replicate 5	12.73	10.61
Replicate 6	12.56	11.33
Replicate 7	12.51	-
Replicate 8	11.03	-
Replicate 9	12.33	
Mean	12.229	11.253
Standard Deviation	0.506	0.500
Coefficient of Variation (%)	4.14	4.44

## Data Availability

The original contributions presented in this study are included in the article. Further inquiries can be directed to the corresponding author.

## Abbreviations

The following abbreviations are used in this manuscript:

BFDV	Beak and Feather Disease Virus
Cq	Cycle Quantification
CV	Coefficient of Variation
DNA	Desoxirribonucleic Acid
LOD	Limit of Detection
LOQ	Limit of Quantitation
NPV	Negative Predictive Value
PBFD	Psittacine Beak and Feather Disease
PCR	Polymerase Chain Reaction
PPV	Positive Predictive Value
RT-PCR	Reverse Transcriptase Polymerase Chain Reaction
